# [OPy][BF_4_] Selective Extraction for Trace Hg^2+^ Detection by Electrochemistry: Enrichment, Release and Sensing

**DOI:** 10.3390/mi12121461

**Published:** 2021-11-27

**Authors:** Chenyu Xiong, Yun Hui, Ri Wang, Chao Bian, Yuhao Xu, Yong Xie, Mingjie Han, Shanhong Xia

**Affiliations:** 1 State Key Laboratory of Transducer Technology, Aerospace Information Research Institute, Chinese Academy of Sciences, Beijing 100190, China; xiongchenyu16@mails.ucas.ac.cn (C.X.); wangri17@mails.ucas.ac.cn (R.W.); cbian@mail.ie.ac.cn (C.B.); xuyuhao15@mails.ucas.ac.cn (Y.X.); xieyong16@mails.ucas.ac.cn (Y.X.); mjhan1993@outlook.com (M.H.); 2 School of Electronic, Electrical and Communication Engineering, University of Chinese Academy of Sciences, Beijing 100049, China; 3 Shenzhen Institutes of Advanced Technology, Chinese Academy of Sciences, Shenzhen 518055, China; huiyunccc@126.com

**Keywords:** mercury ions detection, electrochemical sensor, ionic liquid, liquid–liquid extraction

## Abstract

Trace mercury ion (Hg${}^{2+}$) detection is important for environmental monitoring and water safety. In this work, we study the electrochemical strategy to detect trace Hg${}^{2+}$ based on the preconcentration of temperature-controlled N-octylpyridinium tetrafluoroborate ([OPy][BF${}_{4}$]) dispersive liquid–liquid microextraction. The [OPy][BF${}_{4}$] selectively extracted Hg${}^{2+}$ from aqueous solution by the adsorption of unsaturated N in [OPy][BF${}_{4}$], which increased the concentration of Hg${}^{2+}$ and decreased that of other interference ions. It was noted that the adsorption of [OPy][BF${}_{4}$] to Hg${}^{2+}$ was weakened by aqueous solution. Hence, after extraction, precipitated [OPy][BF${}_{4}$] was diluted by a buffer solution comprising a mixture of water and acetonitrile to release Hg${}^{2+}$ and the single was detected by electrochemistry. Water is proposed to release Hg${}^{2+}$ adsorbed by [OPy][BF${}_{4}$], and the acetonitrile serves as a co-solvent in buffer solution. Sensitivity and anti-inference ability of sensors were improved using the proposed method and Hg${}^{2+}$ releasing procedure. The detection limit (S/N = 3) of the sensor is 0.0315 μg/L with a linear range from 0.1 to 1 μg/L. And the sensor exhibits good recovery with an range from 106 % to 118%, which has great potential for trace Hg${}^{2+}$ determination.

## 1. Introduction

Mercury exhibits high biotoxicity and is a major threat to ecosystems and human health [1] owing to its extremely high bioaccumulation factor in the food chain [2]. Considering the toxicological effects and wide applications of mercury, it is necessary to detect trace Hg${}^{2+}$ in water for ensuring safety. Atomic fluorescence spectrometry (AFS) [3], cold vapor atomic absorption spectrometry (CV-AAS) [4] and inductively coupled plasma mass spectrometry (ICP-MS) [5,6] are authoritative and popular methods for trace mercury detection. However, these methods require large, expensive instrumentation and complex operations. Recently, several simple and novel strategies have been studied and reported for Hg${}^{2+}$ detection, such as colorimetry [7,8], fluorimetry [9,10,11], Raman spectrometry [12,13,14] and localized surface plasmon resonance [15], but most of these methods have low sensitivity, specific labeling and high-cost-instrument requirements. However, Electrochemistry [16,17,18,19] has advantages such as economy, portability and label-free methodology, which is promising for trace mercury detection.

Improving the anti-interference ability of trace Hg${}^{2+}$ electrochemical sensors has increasingly attracted research interest in recent times. In this respect, a popular strategy is to modify the electrode using materials with specific functional groups that bind to mercury, such as metal-organic frameworks [20], metal nanomaterials [21] and thymine [22,23]. However, the application prospects for the electrode modifications method are limited by poor stability and consistency of modification. As an alternative, selective extraction and separation of Hg${}^{2+}$ from the sample not only increases the available concentration of Hg${}^{2+}$ to promote sensitivity of the sensor, but also separates other metal ions to improve the anti-interference ability. However, selective extractants often combine with the Hg${}^{2+}$ by chelation, complexation and similar methods, which limit the electrochemical activity of the Hg${}^{2+}$. Back extraction or releasing the Hg${}^{2+}$ from the extractant is key to achieving trace detection by the combination of extraction and electrochemistry.

Ionic liquids (ILs) have advantages such as wide potential windows and negligible vapor pressure, and have increasingly attracted attention in analytical chemistry [24,25] and electrochemistry [26,27,28] for electrodeposition, electrosynthesis, electrocatalysis and lithium batteries. Mercury ions have been shown to be efficiently extracted or adsorbed by ILs [29]. Our group investigated the abilities of four ILs with N-octylpyridinium cations to selectively capture mercury ions based on the dispersive liquid–liquid microextraction method [16]. Among the four ILs, [OPy][BF${}_{4}$], which is a water-insoluble IL, was found to be an efficient and selective material for capturing Hg${}^{2+}$. However, the mechanism of interaction between [OPy][BF${}_{4}$] and Hg${}^{2+}$ is still not clear. Furthermore, the strategy of releasing the Hg${}^{2+}$ from the [OPy][BF${}_{4}$] needs to be studied, which is the key to realize the trace Hg${}^{2+}$ detection by electrochemistry.

In this work, the temperature-controlled IL dispersive liquid–liquid microextraction (TC-IL-DLLME) technique was used to pre-concentrate the Hg${}^{2+}$ from water owing to its advantages of high speed and the high enrichment factor. We observe that the unsaturated N in [OPy][BF${}_{4}$] can adsorb the Hg${}^{2+}$, therefore effectively and selectively extracting it from an aqueous solution. We also note that the adsorption is weakened by the aqueous solution, therefore, the IL ([OPy][BF${}_{4}$]) enriched Hg${}^{2+}$ was diluted with a buffer solution containing a mixture of water and acetonitrile to improve the electrochemical activity of Hg${}^{2+}$, water was used to weaken the adsorption of IL to Hg${}^{2+}$ and improve its electrochemical activity. Finally, Hg${}^{2+}$ was detected by the differential pulse stripping voltammetry (DPSV), and the detection limit (S/N = 3) was determined as 0.0315 μg/L with a linear range from 0.1 to 1 μg/L. The ultra-trace detection of mercury in water samples exhibits satisfactory anti-interference against various other ions and high recovery. The proposed TC-IL-DLLME-EC strategy is therefore expected to be of great potential for the construction of ultrasensitive electrochemical sensors to detect Hg${}^{2+}$.

## 2. Materials and Methods

### 2.1. Regents and Instruments

[OPy][BF${}_{4}$] was purchased from Shanghai Chenjie Chemical Co, Ltd. (Shanghai, China) Mercury standard stock solution (100 mg/L Hg${}^{2+}$ with 3% nitric acid) was purchased from the China National Research Centre for Certified Reference Material (Beijing, China). Acetonitrile (ACN, 99%) was purchased from Aladdin Chemistry Co., Ltd. (Shanghai, China). Hydrochloric acid (HCl) was purchased from Beijing Chemical Works (Beijing, China). Deionized water was provided by Millipore DQ${}_{3}$UV (Millipore Company, Darmstadt, Germany).

The UV SP-752 spectrophotometer (Tokyo, Japan) was used for photoluminescence spectra. The Zonkia HC-3018 high-speed centrifuge (Hefei, China) was used for centrifugation and separation. Bruker-500 Nuclear Magnetic Resonance Spectrometer (Zurich, Switzerland) was used for 1hydrogen nuclear magnetic resonance analysis. The mercury concentrations in tap water were examined with atomic fluorescence spectrometry by the PONY Testing International Group (PONY Company, Beijing, China). All the electrochemical experiments were carried out on the Gamry Reference 600 electrochemical workstation (Gamry Instruments Co., Ltd., Philadelphia, PA, USA) with the three-electrode system. The gold disc (1 mm diameter) electrode, Platinum electrode and Ag/AgCl (3 M KCl) were used as working, counter and reference electrodes, respectively. Unless otherwise specified, all experiments are carried out at room temperature (25 °C)

### 2.2. Procedures for TC-DLLME and Separation

**Figure 1:**
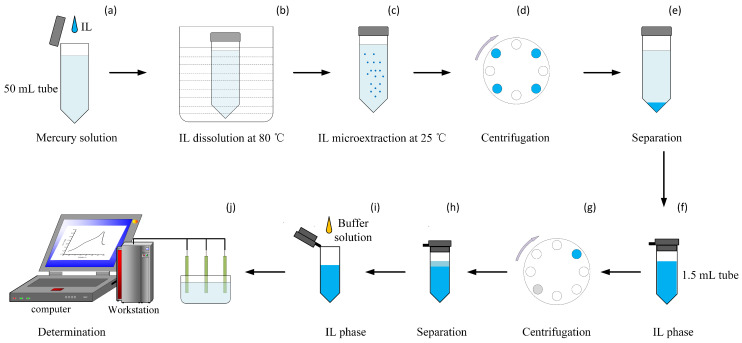
Graphical illustration of the detection of mercury. (**a**,**b**) The mercury dissolved IL achieving saturated IL aqueous solution at 80 °C; (**c**) then temperature decreased to 25 °C with IL droplets suspending in the emulsion. After (**d**,**e**) centrifugation and separation, (**f**) the IL was moved into a 1.5 mL tube. After (**g**,**h**) centrifugation and separation again, (**i**) the IL phase was mixed with ACN and water solution containing the HCl. (**j**) Mercury ions were detected by DPSV. ((**a**–**h**) the process of TC-DLLME, (**i**,**j**) electrochemical detection).

The TC-IL-DLLM technique was adopted to pre-concentrate the Hg${}^{2+}$ from water owing to its advantage of reducing times and the high enrichment factor [30]. As shown in Figure 1a–h. First, 4 mL [OPy][BF${}_{4}$] added into 180 mL water sample at 80 °C in the 4*50 mL centrifuge tube (Figure 1a). Then the mixture was continuously shaken until the IL dissolved completely, and the 50 mL centrifuge tube with the mixture was heated in a water bath at 80 °C for 5 min (Figure 1b). Then the tube containing the mixture was cooled to room temperature, several fine [OPy][BF${}_{4}$] droplets with enriched Hg${}^{2+}$ were hence formed and dispersed in the solution due to low solubility in water at 25 °C (Figure 1c). The mixture was separated into the aqueous and the IL phase after centrifugation at 3000 rpm for 10 min (Figure 1d). Then the supernatant was removed, and IL phase retained, and the IL phase was placed in 1 mL centrifuge tubes and centrifugated at 3000 rpm, with removal of the supernatant to ensure full removal of the water in the mixture(Figure 1e–h).

### 2.3. Electrochemical Detection

For the detection, the IL phase with enriched Hg${}^{2+}$ was dissolved in the mixture of water and acetonitrile (ACN) to release Hg${}^{2+}$. The final test solution with 0.1 M hydrochloric acid (HCl) was made of [OPy][BF${}_{4}$], acetonitrile and deionized water (volume ratio of 1:1.8:1.2). Then, DPSV was adopted to detect Hg${}^{2+}$ in the test solution. Different voltages were first applied to the working electrode, 1 V for 60 s to clean the electrode surface. The potential was maintained at 0.1 V for 360 s, the Hg${}^{2+}$was reduced to Hg${}^{0}$ and was enriched around surface. Then the potential of the working electrode was scanned from 0.1 V to 0.7 V to detect Hg${}^{2+}$ with a step size of 4 mV, sample period of 0.1 s, pulse time of 0.02 s and pulse size of 50 mV. When potential scanned from 0.1 V to 0.7 V, Hg${}^{0}$ was stripped from the electrode surface and oxidized to Hg${}^{2+}$. The current response curve was then obtained with an oxidation peak at 0.50 V. Each measurement was repeated at least three times.

### 2.4. Procedures for Mercury Detection Based on TC-DLLME

As depicted in Figure 1, the overall detection procedures include TC-DLLME (Figure 1a–c) and electrochemical detection (Figure 1i,j). Different concentrations of the mercury solution were obtained by diluting the mercury standard solution stepwise with a concentration of 100 μg/L. After TC-DLLME, the 600 μL [OPy][BF${}_{4}$] with Hg${}^{2+}$ was obtained. Then 500 μL of the [OPy][BF${}_{4}$] was added to 1.5 mL of the buffer solution, which was composed of 0.6 mL H${}_{2}$O (0.4 mL deionized water, 0.2 mL 1M HCl) and 0.9 mL ACN (Figure 1i). The mixture was fully shaken and used as the test solution. Next, the concentrations of mercury ions from among the samples were detected by stripping voltammetry (Figure 1j).

## 3. Results

### 3.1. Preconcentration

#### 3.1.1. The Enrichment Factor

The TC-IL-DLLME method, a rapid microextraction of an immiscible IL from water, has several advantages such as high speed and high enrichment factor (EF). The higher enrichment factor represents higher concentration of the mercury in the final IL phase, which is beneficial for detection of trace concentrations. The enrichment factor is defined as the ratio of the initial mercury concentration in water to the ultimate mercury concentration in the IL phase, which is depicted as in Formula (1), where C${}_{ult}$ refers to the ultimately extracted mercury concentration in the IL phase, and C${}_{in}$ refers to the concentration of mercury in the initial aqueous solution. The value of C${}_{ult}$ is calculated using Formula (2), where C${}_{fin}$ is the final concentration of mercury in the aqueous phase after preconcentration. V${}_{aq}$ refers to the initial volume of the aqueous phase and V${}_{IL}$ refers to the final volume of the IL phase. Therefore, the EF is calculated using Formula (2). The value of C${}_{in}$ and C${}_{fin}$ are measured by AFs. The average EF is calculated as 342 over several minutes.

(1) $$EF=\frac{C_{ult}}{C_{in}}$$

(2) $$C_{ult}=\left(C_{in}-C_{fin}\right)\times \frac{V_{aq}}{V_{IL}}$$

(3) $$EF=\frac{\left(C_{in}-C_{fin}\right)}{C_{in}}\times \frac{V_{aq}}{V_{IL}}$$

#### 3.1.2. Mechanism

During preconcentration, Hg${}^{2+}$ was effectively and selectively enriched by [OPy][BF${}_{4}$] from water containing other interfering ions [16]. However, a clear interaction between [OPy][BF${}_{4}$] and Hg${}^{2+}$ is still not observed, which is studied in the following experiments; meanwhile, the strategy for improving the electrochemical activity of Hg${}^{2+}$ was proposed and proved.

**Figure 2:**
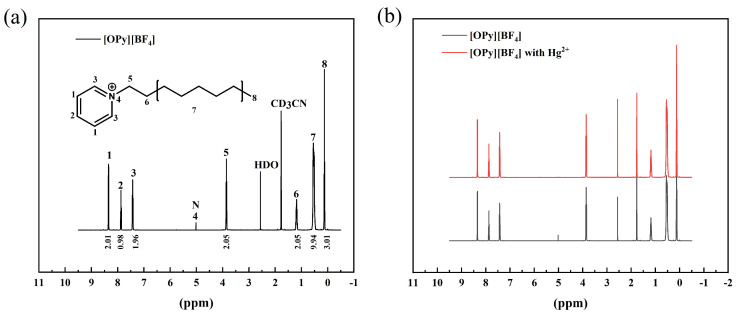
(**a**) The H-NMR of [OPy][BF${}_{4}$]; (**b**) the H-NMR of [OPy][BF${}_{4}$] with 10 μM Hg${}^{2+}$ and pure [OPy][BF${}_{4}$]. The [OPy][BF${}_{4}$] was dissolved in deuterated acetonitrile (CD${}_{3}$CN).

To investigate the extraction mechanism, H-NMRs of pure [OPy][BF${}_{4}$] and [OPy][BF${}_{4}$] with Hg${}^{2+}$ were measured and compared. First, 10 μL of [OPy][BF${}_{4}$] with 10 μM Hg${}^{2+}$ and pure [OPy][BF${}_{4}$] were added to 1mL deuterated acetonitrile (CD${}_{3}$CN). Then, the two samples were analyzed at the Peking University Testing and Analysis Center. Figure 2a shows the H-NMR of [OPy][BF${}_{4}$], a weak hydrogen peak is seen at a chemical shift (δ) of 5.2. This weak peak indicates that unsaturated nitrogen has interacted with intermolecular hydrogen. Compared with [OPy][BF${}_{4}$], the hydrogen peak of the mercury-containing [OPy][BF${}_{4}$] disappears where the δ is 5.2, indicating that N could adsorb the Hg${}^{2+}$.

Ionic liquids with octylpyridine ions can selectively extract mercury, in which the pyridine ring plays a major role, and the phenomenon is affected by anions [29]. Besides the Coulomb force, the hydrogen bond and network structure are another important noncovalent interaction in the IL and are closely related to some important properties [31]. In this work, unsaturated N is positively charged, so there was electrostatic repulsion and adsorption between unsaturated N and Hg${}^{2+}$. The selective extraction indicated that the adsorption effect is stronger, and the anions will also enter the ionic liquid to balance the electrostatic effect and maintain electrical neutrality. Therefore, stable molecular clusters were formed through the interaction of ionic liquids, Hg${}^{2+}$ and anions.

**Figure 3:**
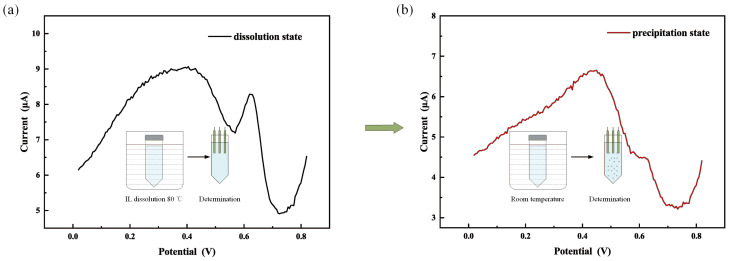
Solution appearance and DPSV signal of mercury during TC-DLPME; (**a**) IL is completely dissolved in the sample; (**b**) IL is separated from the sample. The initial solution contains 100 μgL${}^{-1}$ Hg${}^{2+}$ and deposited potential was 0 V, and the deposited time was the 60 s.

To investigate the process of adsorption of Hg${}^{2+}$ during TC-IL-DLLME, the electrochemical signal of Hg${}^{2+}$ was monitored during extraction by DPSV. As shown in Figure 3, a current peak of mercury was observed when [OPy][BF${}_{4}$] was completely dissolved in the sample, indicating that [OPy][BF${}_{4}$] hardly captures Hg${}^{2+}$ when dissolved in water. However, the electrochemical signal of Hg${}^{2+}$ disappeared when [OPy][BF${}_{4}$] droplets were formed and dispersed in the mixture, which indicated that the ability to capture Hg${}^{2+}$ is strong when [OPy][BF${}_{4}$] is separated from the water phase. These results show that water weakens the combination of [OPy][BF${}_{4}$] and Hg${}^{2+}$.

From another perspective, when the ionic liquid was precipitated, the ionic liquid precipitated was electrically neutral, unsaturated N adsorbs mercury ions, a stable molecular cluster structure was formed. When the ionic liquid was completely dissolved in water, the molecular cluster structure was destroyed, which may be caused by active protons from the water attacked the molecular cluster structure. The stable state was destroyed, IL cannot bind to Hg${}^{2+}$ due to electrostatic repulsion force between N and Hg${}^{2+}$.

### 3.2. The Electrochemical Activity of Hg${}^{2+}$

**Figure 4:**
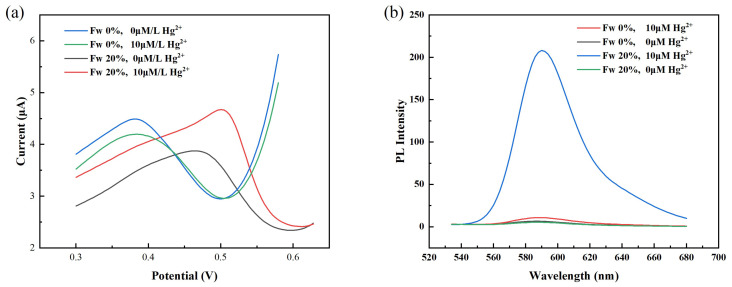
(**a**) the current response curve in ACN buffer and the buffer of water and ACN respectively; (**b**) the PL Intensity curve in ACN buffer and the buffer of water and ACN, respectively. ( Fw = V${}_{H_{2}O}$: V${}_{H_{2}O}$ + V${}_{ACN}$).

The extraction by [OPy][BF${}_{4}$] was consistent in the presence of 1 M hydrochloric, sulfuric, nitric and perchloric, which indicated mercury cannot be back extracted into aqueous phase then was detected by the common method of increasing proton intensity of aqueous phase [32,33]. Based on the observation that water weakens the combination of [OPy][BF${}_{4}$] and Hg${}^{2+}$, the strategy for improving the electrochemical activity of Hg${}^{2+}$ is proposed. The [OPy][BF${}_{4}$] is first dissolved in a buffer solution of water and acetonitrile because the [OPy][BF${}_{4}$] is insoluble in water at room temperature. As shown in Figure 4a, when [OPy][BF${}_{4}$] with Hg${}^{2+}$ is dissolved in the buffer solution of water and acetonitrile, an obvious Hg${}^{2+}$ current peak appears near 0.5 V; however, when acetonitrile is used as the buffer solution, there is almost no signal peak of Hg${}^{2+}$. The added water promotes the response of the sensor to Hg${}^{2+}$. It indicated the addition of water weakens the adsorption of N in [OPy][BF${}_{4}$] to Hg${}^{2+}$, which caused by the addition of water destroyed the molecular cluster structure. Thus, releasing Hg${}^{2+}$ from [OPy][BF${}_{4}$] and enabling its detection by electrochemistry.

To verify the state of mercury in the test solution, an ionic Hg${}^{2+}$ probe [10] was used to detect the amount of Hg${}^{2+}$ in the acetonitrile buffer and the buffer solution of water and acetonitrile. First, the [OPy][BF${}_{4}$] with Hg${}^{2+}$ was mixed with the two buffers containing the probes; then, the test sample was heated and incubated at 55 °C for 5 h. Finally, the sample was analyzed using a fluorescence spectrophotometer. The other experimental details are reported in a recent paper [9]. The corresponding fluorescence curves of the test samples in different buffers are shown in Figure 4b. The added water increases the concentration of Hg${}^{2+}$ in the solution, but when acetonitrile is used as the buffer, mercury is rarely in the form of Hg${}^{2+}$ in the test sample. These experimental results confirm that the added water could promote the release of Hg${}^{2+}$ and effectively improved the electrochemical activity of Hg${}^{2+}$. Therefore, the mixture of water and ACN was chosen as the buffer solution for releasing Hg${}^{2+}$.

### 3.3. Optimization of Electrochemical Detection

Parameters of effecting the Hg${}^{2+}$ response were studied, including the volume ratios of IL, H${}_{2}$O and ACN and deposition time. In the experiments, the IL with the solid HgCl${}_{2}$ (5 μM) served as the replacement for the separated IL with enriched Hg${}^{2+}$, and ILs with different concentrations of Hg${}^{2+}$ were prepared by careful stepwise dilution.

#### 3.3.1. Effect of Volume Ratios of IL, H${}_{2}$O and ACN

**Figure 5:**
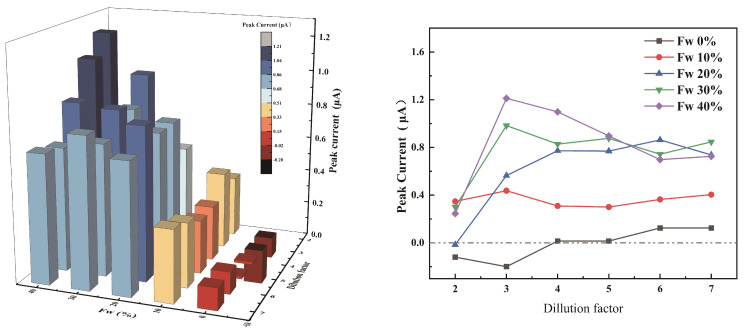
Effect of the detection solution system. The deposition potential was 0.1 V and the deposition time was 240 s.

The effect of different volume ratios of IL, H${}_{2}$O and ACN was studied by introducing two parameters, namely water fraction (Fw) and dilution factor (DF). The water fraction indicates the volume percent of water in the buffer solution, and the dilution factor refers to the volume ratio of the IL compared to the total volume. Figure 5 shows the current response peaks of Hg${}^{2+}$ after removing the current peaks of blank solutions (ILs without Hg${}^{2+}$) for different buffer systems. When the water fraction (Fw) is 0, the current response of Hg${}^{2+}$ is low, because the mercury in the test solution is not released. When water is added, the current response increases obviously, the main reason for this is that the added water breaks the adsorption effect of IL to Hg${}^{2+}$. The current peak tends to increase as Fw increased, and the reason for this is the higher Fw allows the mercury restricted by the IL to result in more free Hg${}^{2+}$. Based on the results in Figure 5, the condition with Fw of 40% and dilution factor of 3 replaced the best condition with Fw of 40% and dilution factor of 2, this configuration was used in the following experiments based on consideration of the final test solution volume and initial water sample volume.

(4) $$DF=\frac{V_{IL}+V_{H_{2}O}+V_{ACN}}{V_{IL}}$$

(5) $$F_{W}=\frac{V_{H_{2}O}}{V_{H_{2}O}+V_{ACN}}$$

#### 3.3.2. Effect of Deposition Time

**Figure 6:**
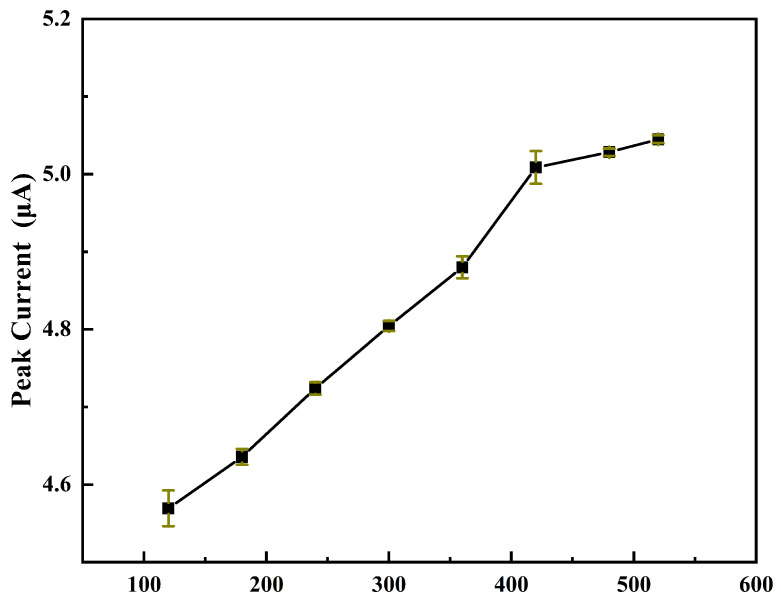
The current peak of Hg${}^{2+}$ in different deposition time.

The influence of the pre-deposition time on the Hg${}^{2+}$ string response is studied in the range from 60 s to 520 s. As depicted in Figure 6, we choose 420 s for the potential time because the peak current growth slows after 420 s.

### 3.4. Analytical Performance

#### 3.4.1. Electrochemistry Based on Microextraction for the Detection

**Figure 7:**
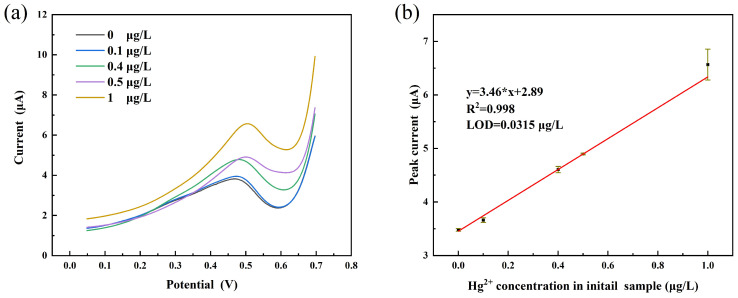
(**a**) current response of different Hg${}^{2+}$ concentrations; (**b**) liner response curve to Hg${}^{2+}$.

The initial samples were prepared with 0 μg/L, 0.1 μg/L, 0.4 μg/L, 0.5 μg/L, and 1 μg/L of Hg${}^{2+}$ solution in deionized water. The response curves for these solutions are shown in Figure 7a, and the linear curves are shown in Figure 7b. The linear response range is observed from 0.1 μg/L to 1 μg/L with a correlation coefficient of 0.998; further, the limit of detection (LOD) is 0.315 μg/L (S/N = 3). This result indicates that the electrochemical sensor can be used for the detection of trace Hg${}^{2+}$ in aqueous solutions.

#### 3.4.2. Anti-Interference Research

**Figure 8:**
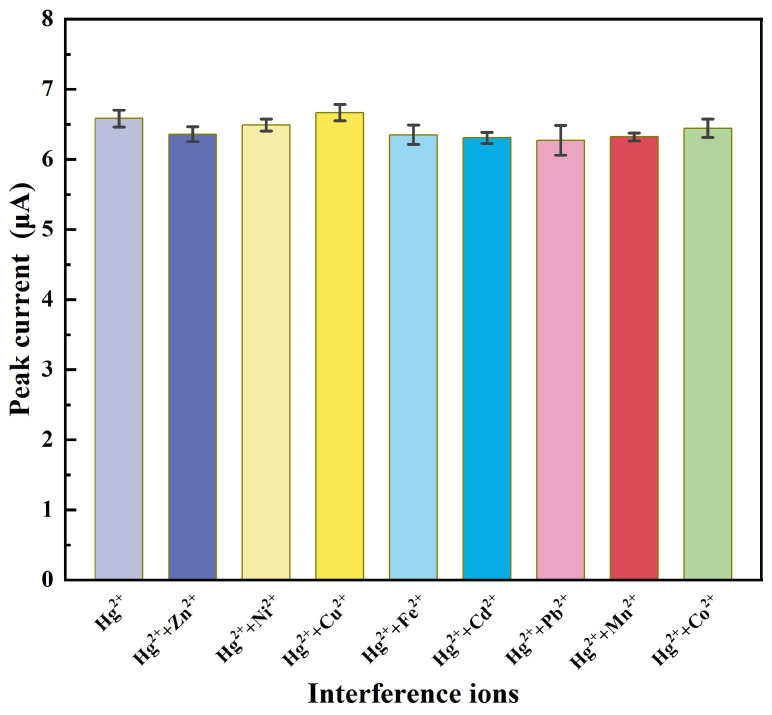
Current peak in the presence of 10 μg/L marked metal ions followed by the addition of 1 μg/L Hg${}^{2+}$ with the blank solution subtracted.

The effects of coexisting metal cations in the water samples were studied along with mercury detection. The ions Zn${}^{2+}$, Ni${}^{2+}$, Cu${}^{2+}$, Fe${}^{2+}$, Cd${}^{2+}$, Pb${}^{2+}$, Mn${}^{2+}$, and Co${}^{2+}$ were chosen for the anti-interference tests against Hg${}^{2+}$ because these are heavy metal cations that have similar properties to Hg${}^{2+}$. First, these test samples with 1 μg/L Hg${}^{2+}$ and other interference ions were prepared. After TC-DLLME, the IL was gained, and the 0.5 IL was added to 1.5 mL of the buffer solution. Finally, the test solution was detection by DPSV. As shown in Figure 8, the peak current does not change significantly with the additional 10× concentrations of the other interference metal cations; this is attributed to the removal of the interfering ions during the pretreatment process. These results indicate that the test method has good anti-interference ability for mercury detection.

#### 3.4.3. Test in the Tap Water

To investigate the potential for detection of ultra-trace Hg${}^{2+}$ in real samples, tap water was collected from our lab and analyzed under optimal conditions. Furthermore, this water was spiked with 0.3 μg/L, 0.5 μg/L, and 1 μg/L of the Hg${}^{2+}$ standard solution, and the concentrations of Hg${}^{2+}$ are certified by AFS and the proposed method. These results are described in Table 1. The proposed method shows satisfactory results in a range of 106% to 118% for detection of water samples.

#### 3.4.4. Comparison of Analytical Performance with Other Reports

Our method adopts the three-electrode system, with a simple gold electrode as the working electrode. Our proposed work mainly focuses on realizing detection of mercury by anodic stripping analysis. Analytical performances are compared with several reported methods of trace mercury determination using anodic stripping, as shown in Table 2. Our work obtains higher concentrations of the samples by preconcentration and has lower detection limits, as shown in the table.

## 4. Conclusions

In this work, we report an electrochemical approach for trace Hg${}^{2+}$ detection based on TC-IL-DLLME. The [OPy][BF${}_{4}$] can be used to effectively and selectively enrich Hg${}^{2+}$ from water samples, therefore improving the concentration of Hg${}^{2+}$ and reducing the concentrations of other interference ions. We have observed that there are important interactions between [OPy][BF${}_{4}$] and Hg${}^{2+}$, the unsaturated N in [OPy][BF${}_{4}$] can adsorb Hg${}^{2+}$ while the adsorption can be weakened by water. Based on this mechanism, the strategy for improving the electrochemical activity of Hg${}^{2+}$ is proposed and demonstrated. Owing to the effective and selective enrichment of Hg${}^{2+}$, the method developed in this work provides a higher enrichment factor, greater selectivity, and a lower limit of detection. Moreover, the ultra-trace detection of mercury in water samples exhibit good recovery. Thus, the proposed electrochemical method based on the TC-IL-DLLME-EC strategy is expected to have great potential for the construction of ultrasensitive electrochemical sensors for the on-site determination of Hg${}^{2+}$ in water.

## Figures and Tables

**Table 1:** Analytical result by the TC-IL-DLLME-DPSV method in tap water.

Tap Water	Added (μg/L)	AFS (μg/L)	Detected (μg/L)	Recovery
Tap water 1	0.3	0.31 ± 0.01	0.32 ± 0.02	106%
Tap water 2	0.5	0.53 ± 0.01	0.58 ± 0.07	116%
Tap water 3	1	1.09 ± 0.01	1.18 ± 0.08	118%

**Table 2:** Comparison of analytical performance for the determination of trace mercury in the aqueous sample.

Electrode	Method	LOD (μg/L)	Reference
BieAuNPs/CPE	SWASW	0.3	[34]
SePs-AuNPs/CPE	DPSV	1.02	[18]
Fe3O4/DNA/GCE	DPSV	0.066	[19]
Gold electrode	DPSV	0.031	This work
